# Head-to-Head Comparison of Transcranial Random Noise Stimulation, Transcranial AC Stimulation, and Transcranial DC Stimulation for Tinnitus

**DOI:** 10.3389/fpsyt.2013.00158

**Published:** 2013-12-18

**Authors:** Sven Vanneste, Felipe Fregni, Dirk De Ridder

**Affiliations:** ^1^Department of Translational Neuroscience, Faculty of Medicine, University of Antwerp, Antwerp, Belgium; ^2^Laboratory of Neuromodulation, Department of Physical Medicine and Rehabilitation, Spaulding Rehabilitation Hospital and Massachusetts General Hospital, Harvard Medical School, Boston, MA, USA; ^3^School of Behavioral and Brain Sciences, University of Texas at Dallas, Dallas, TX, USA; ^4^Center for Clinical Research Learning, Harvard Medical School, Boston, MA, USA; ^5^Department of Surgical Sciences, Dunedin School of Medicine, University of Otago, Otago, New Zealand

**Keywords:** tDCS, tACS, tRNS, tinnitus, loudness, distress

## Abstract

Tinnitus is the perception of a sound in the absence of an external sound stimulus. This phantom sound has been related to plastic changes and hyperactivity in the auditory cortex. Different neuromodulation techniques such as transcranial magnetic stimulation and transcranial direct current stimulation (tDCS) have been used in an attempt to modify local and distant neuroplasticity as to reduce tinnitus symptoms. Recently, two techniques of pulsed electrical stimulation using weak electrical currents – transcranial alternating current stimulation (tACS) and transcranial random noise stimulation (tRNS) – have also shown significant neuromodulatory effects. In the present study we conducted the first head-to-head comparison of three different transcranial electrical stimulation (tES) techniques, namely tDCS, tACS, and tRNS in 111 tinnitus patients by placing the electrodes overlying the auditory cortex bilaterally. The results demonstrated that tRNS induced the larger transient suppressive effect on the tinnitus loudness and the tinnitus related distress as compared to tDCS and tACS. Both tDCS and tACS induced small and non-significant effects on tinnitus symptoms, supporting the superior effects of tRNS as a method for tinnitus suppression.

## Introduction

Tinnitus is an auditory phantom phenomenon of a sound perception in the absence of an objective physical sound source (1). Tinnitus affects 5–15% of the western population and between 6 and 25% of the affected people report symptoms that are severely debilitating (2). Between 2 and 4% of the whole population suffers in the worst degree, leading to a noticeable decrease in the quality of life (3). Psychological complications such as lifestyle detriment, emotional difficulties, sleep deprivation, work hindrance, interference with social interaction, and decreased overall health have been attributed to tinnitus (4).

Analogous to phantom pain, tinnitus is also considered an auditory phantom percept related to plastic changes in the auditory cortex (5, 6), resulting from a filling-in mechanism associated with auditory deafferentation (7). Neuroimaging and electrophysiological studies indicate that excessive spontaneous activity in the central auditory nervous system and changes in the tonotopic map of the auditory cortex are associated with the presence of tinnitus (5, 8–11). These data are in accordance with the thalamo-cortical dysrhythmia model that proposes that tinnitus is caused by an abnormal, spontaneous, and constantly coupled persisting theta/gamma band activity generated as a consequence of hyperpolarization of specific thalamic nuclei, in casu the medial geniculate body. In normal circumstances auditory stimuli increase thalamo-cortical rhythms to gamma band activity (12). In the deafferented state however, oscillatory activity decreases from alpha activity to theta band activity (13). As a result lateral inhibition is reduced inducing a surrounding gamma band activity known as the “edge effect” (14, 15). Indeed, a strong inverse relationship between alpha and gamma power in tinnitus patients has been shown (16) and the perceived tinnitus loudness is correlated to increased gamma band activity in the auditory cortex (17). Furthermore, in a tinnitus patient with an implanted electrode overlaying the auditory cortex increased gamma (>30 Hz) and theta peaks (4–7 Hz) were measured, and the theta and gamma activity was coupled (18). Interestingly this mechanism is similar to neuropathic pain, including phantom limb pain, in which a neural lesion leads to increased thalamo-cortical activity as supported by studies in neuropathic pain showing decreased intracortical inhibition (19, 20).

Given the mechanism of central maladaptive plasticity associated with sensory deafferentation, it has been proposed that interfering with this pathological thalamo-cortical activity is possible, both with invasive (18, 21, 22) and non-invasive neuromodulation (23, 24). Non-invasive neuromodulation techniques such as transcranial magnetic stimulation (TMS) (23, 25–31) and transcranial direct current stimulation (tDCS) (27, 31–33) have emerged as interesting and promising techniques for modulating tinnitus related activity (23). Recently, transcranial alternating current stimulation (tACS) and transcranial random noise stimulation (tRNS) have been developed as novel neuromodulatory devices. These three techniques can be considered as different forms of transcranial electrical stimulation (tES), each with a different working mechanism.

Depending on the polarity of the stimulation, tDCS can increase or decrease cortical excitability in the brain regions to which it is applied (34). Currently, tDCS is usually applied through two surface electrodes, one serving as the anode and the other as the cathode, with the current flowing constantly from the anode to the cathode (35). Some of the applied current is shunted through scalp tissue and only a part of the applied current passes through the brain (36). Anodal tDCS typically exerts an excitatory effect on the local cerebral cortex by depolarizing neurons, while under the cathode hyperpolarization is induced; though the final effects of anodal and cathodal tDCS also depends on other parameters such as baseline cortical activity (37). These effects of tDCS typically outlast the stimulation by an hour or longer after a single treatment session of sufficiently long stimulation duration (38).

Another technique that has also been given more recent attention is tACS which also is potentially capable of interacting with rhythmic neuronal activity and has perceptual and behavioral consequences (39). This method relies on application of alternating currents through an electrode and is no longer sensitive to the direction of current flow. Electrical currents are applied constantly at low intensities over a period of time and allow manipulation of intrinsic cortical oscillations with externally applied electrical frequencies. As such, tACS is better suited to modulate functions that are closely related to brain oscillations at specific frequencies (40). For example, tACS strengthens the individual alpha frequency (IAF) of the stimulated area (40). Also, recent computer modeling data has shown that pulsed AC stimulation induces significant electrical fields in subcortical areas (41); thus potential differences between techniques of electrical stimulation may be due to differences in the induced electrical current fields.

Another method that has also been tested more is tRNS. This method includes a normally distributed random level of current generated with a frequency spectrum between 0.1 and 640 Hz at a sampling rate of 1280 samples per second with no overall DC offset. The frequency spectrum looks similar to the “white noise” characteristic. Research showed that tRNS has a consistent excitability increase lasting at least 60 min, both on physiological and behavioral measures (42). Long-term potentiation has been postulated as a likely mechanism underlying these effects (43). It was furthermore suggested that the mechanism of action of tRNS was based on repeated subthreshold stimulations, which may prevent homeostasis of the system and potentiate task-related neural activity (44).

Many groups have studied and reviewed the neurophysiological and clinical effects of tES with direct current in tinnitus (27, 31–33, 45–49). Less effort has been dedicated to the study of stimulation with alternating current stimulation or random noise stimulation. So far, no studies have examined the clinical effect of tACS and tRNS in tinnitus. As tACS can strengthen the IAF (40) and also has shown to increase intracortical inhibition (50), this could theoretically counteract the decreased alpha power that is associated with an increase of theta and gamma power (16, 18) in the auditory cortex according to the thalamo-cortical dysrhythmia model (51). By modulating the alpha frequency it should be possible to modulate the tinnitus percept. Applying tRNS might induce an improvement by potentially disrupting tinnitus related synchrony in the auditory cortex, analogous to what has been proposed by acoustic coordinated reset stimulation (52). In the present study we aim to test the efficacy of tACS as compared to other two different tES techniques on tinnitus – one that has shown significant effects (tDCS) and the other that also uses pulsed AC current but with different parameters of frequency arrangement in a head-to-head trial. We therefore compared the effects of tDCS, tACS, or tRNS applied bilaterally on the auditory cortex.

## Materials and Methods

### Participants

One hundred and eleven tinnitus patients (*N* = 111; 77 females and 34 males) with a mean age of 49.46 (SD = 14.37 years) were selected from the multidisciplinary Tinnitus Research Initiative (TRI) Clinic of the University Hospital of Antwerp, Belgium. Patients had a mean tinnitus duration of 4.18 years (SD = 4.05 years). Table 1 gives an overview of the demographics and tinnitus characteristics. Individuals with pulsatile tinnitus, Ménière disease, otosclerosis, chronic headache, neurological disorders such as brain tumors, and individuals being treated for mental disorders (i.e., neuropsychiatric diseases) were not included in the study in order to obtain a homogeneous sample. All patients had tinnitus for more than 1 year and have a tinnitus that is constantly present. No psychoactive neuropharmaca were added or removed during the trial period in the tDCS, tACS, and tRNS groups.

**Table 1:** **Patients’ demographics and tinnitus characteristics**.

	tDCS		tACS	tRNS	Total
	Anodal left	Anodal right			
Gender
Male	7	5	10	12	34
Female	13	11	27	26	77
Age
Mean	50.05	47.06	49.21	50.39	49.46
SD	14.99	15.07	14.26	14.3	14.37
Tinnitus site
Left-side	3	2	10	8	23
Right-side	5	4	9	13	31
Bilateral	12	10	18	17	57
Tinnitus type
Pure tone	11	8	18	14	51
Narrow band noise	9	8	19	24	60
Tinnitus duration
Mean	4.18	4.18	3.83	4.52	4.18
SD	4.67	3.23	4.23	3.96	4.05
Tinnitus loudness
Mean	6.75	6.5	6.69	7.07	6.8
SD	1.71	1.71	1.68	1.67	1.71
Tinnitus distress
Mean	6.1	6.25	6.86	6.79	6.61
SD	2.12	1.69	1.67	1.7	1.77

Participants were requested to refrain from alcohol consumption 24 h prior to recording and from caffeinated beverages on the day of recording.

This study was approved by the local ethical committee (Antwerp University Hospital) and was in accordance with the declaration of Helsinki. Patients signed a written informed consent before the procedure.

### Transcranial electrical stimulation

For the three conditions of stimulation (tDCS, tACS, and tRNS), we used similar electrode size (35 cm^2^), parameters of stimulation (1.5 mA and 20 min) and location of stimulation (one electrode in T3 and one electrode in T4). For most of subjects, 1.5 mA is under the sensory perception threshold; thus there were no clear differences in perception between these techniques. Subjects were informed that we were comparing three active conditions of tES.

### Transcranial direct current stimulation

Direct current was transmitted by a saline-soaked pair of surface sponge (35 cm^2^) and delivered by specially developed, battery-driven, constant current stimulator with a maximum output of 10 mA (NeuroConn; http://www.neuroconn.de/). For 16 patients receiving tDCS, the cathode was placed over the left auditory cortex and the anode was placed on the right auditory cortex as determined by the International 10/20 Electroencephalogram System, corresponding to T3 and T4 respectively. For 20 patients the cathode was placed over T4 and the anode over T3. The DC current was initially increased in a ramp-like fashion over several seconds (10 s) until reaching 1.5 mA and stimulation was maintained for a total of 20 min.

### Transcranial alternating current stimulation

To determine the frequency of stimulation, the IAF peak was identified according to literature guidelines (53). This IAF peak was defined as the frequency within the range of 6–13 Hz range of the EEG spectrum showing maximum power for the electrodes T3 and T4.

EEGs (Mitsar, Nova Tech EEG, Inc., Mesa) were obtained 1 week before the tACS stimulation in a fully lighted room with each participant sitting upright in a comfortable chair. The EEG was sampled with 19 electrodes (Fp1, Fp2, F7, F3, Fz, F4, F8, T7, C3, Cz, C4, T8, P7, P3, Pz, P4, P8, O1, O2) in the standard 10–20 International placements referenced to linked lobes and impedances were checked to remain below 5 kΩ. Data were collected for 100, 2-s epochs eyes closed, sampling rate = 1024 Hz, and band passed 0.15–200 Hz. Data were resampled to 128 Hz, band-pass filtered (fast Fourier transform filter) to 2–44 Hz. These data were transposed into Eureka! Software (Congedo, 2002),^1^ plotted and carefully inspected manually for artifact. All episodic artifacts including eye blinks, eye movements, teeth clenching, body movement, or ECG artifacts were removed from the stream of the EEG.

Alternating current was transmitted by a saline-soaked pair of surface sponge (35 cm^2^) and delivered by specially developed, battery-driven, constant current stimulator with a maximum output of 10 mA (NeuroConn; http://www.neuroconn.de/). For each patient receiving tACS, one electrode was placed on the T3 and one was placed on T4 as determined by the International 10/20 Electroencephalogram System. The frequency of the tACS was set to the IAF. In both real tACS and sham, the AC current was initially increased in a ramp-like fashion over several seconds (10 s) until reaching 1.5 mA. In tACS, stimulation was maintained for a total of 20 min.

### Transcranial random noise stimulation

The tRNS consisted of an alternating current of 1.5 mA intensity with a 0-mA offset applied at random frequencies. The frequencies ranged from 0.1 to 100 Hz. Similar to tDCS or tACS the current was transmitted by a saline-soaked pair of surface sponge (35 cm^2^) and delivered by specially developed, battery-driven, constant current stimulator with a maximum output of 10 mA (NeuroConn; http://www.neuroconn.de/). For each patient receiving tRNS, one electrode was placed on the T3 and one was placed on T4 as determined by the International 10/20 Electroencephalogram System. The AC current was initially increased in a ramp-like fashion over several seconds (10 s) until reaching 1.5 mA. In tRNS, stimulation was maintained for a total of 20 min.

### Evaluation

Patients were randomly assigned to the tDCS, tACS, or tRNS treatment. Thirty-six patients underwent tDCS (20 anode left auditory cortex and 16 anode right auditory cortex), 37 tACS and 38 patients received tRNS. A numeric rating scale (NRS) for tinnitus intensity (“How loud is your tinnitus? 0 = no tinnitus and 10 = as loud as imaginable”) and tinnitus distress (“How annoying is your tinnitus? 0 = not annoying 10 = suicidal annoying”) was asked before (pre) and directly after (post) stimulation.

### Statistical analysis

#### Calculations were performed using SPSS software package

A repeated measure ANOVA was conducted with evaluation pre-NRS versus post-NRS as the within-subjects variable and type of stimulation (tDCS, tACS, and tRNS) as the between-subjects variables for both distress and loudness in one model. We used simple contrast analyses as this method allows us to test the statistical significance of predicted specific differences in particular parts of our complex design.

To confirm our data we applied a multivariate ANOVA with the subtraction between pre- and post-stimulation for respectively tinnitus loudness and tinnitus distress as dependent variables and the type of stimulation as independent variable.

## Results

A univariate analysis revealed that, for the pre-stimulation time point, there was no significant difference between the three stimulation types on both the tinnitus loudness (*F*(2,108) = 0.72, *p* = 0.49) and tinnitus distress (*F*(2,108) = 1.72, *p* = 0.18).

### Difference between anodal and cathodal stimulation

To verify whether there was a difference between the two tDCS stimulation types (anode left/cathode right versus anode right/cathode left) we conducted a repeated measures ANOVA pre-RNS (NRS) versus post-RNS as the within-subjects variable and as the between-subjects variable for both distress and loudness in one model. This analysis demonstrated that there was no significant effect between both condition (*F*(2,33) = 0.43, *p* = 0.661) on both tinnitus loudness (*F*(1,34) = 0.04, *p* = 0.85) and tinnitus distress (*F*(1,34) = 0.15, *p* = 0.70). Hence we bring both groups together into one larger tDCS group.

### Differences between tDCS, tACS, and tRNS

**Figure 1:**
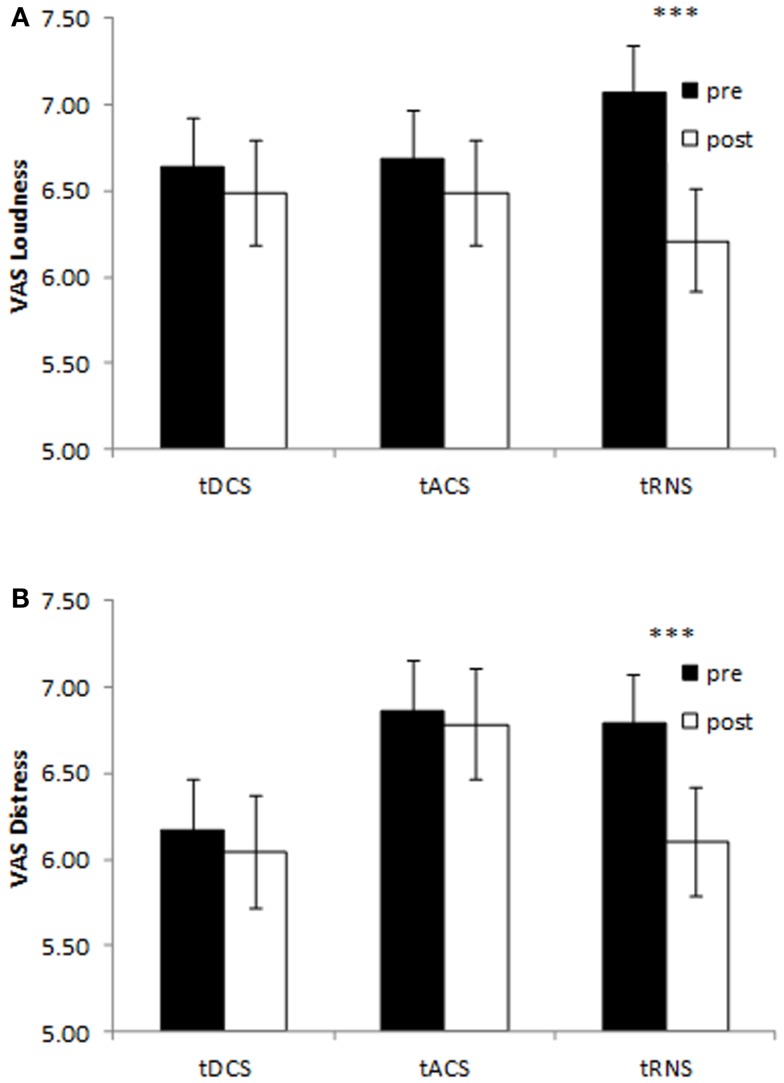
**Pre- and post-stimulation numeric rating scales for tinnitus loudness (A) and tinnitus distress (B) for bilateral auditory cortex tDCS, tACS, and tRNS**. Only tRNS exerts a suppressive effect both tinnitus loudness and tinnitus distress. (***p < 0.001).

A repeated measure ANOVA was conducted with evaluation pre-NRS versus post-NRS as the within-subjects variable and type of stimulation (tDCS, tACS, and tRNS) as the between-subjects variables for both distress and loudness in one model. A main significant effect was obtained between pre-stimulation and post-stimulation measurements (*F*(2,107) = 9.19, *p* = 0.0002) on the both tinnitus loudness (*F*(2,108) = 16.05, *p* = 0.0001) and tinnitus distress (*F* = 9.62, *p* = 0.002). That is, a significant decrease was obtained after stimulation on the tinnitus loudness (*M* = 6.39, SD = 1.83) in comparison with the tinnitus loudness before stimulation (*M* = 6.81, SD = 1.68). A similar effect was obtained for the tinnitus distress indicating a decrease the after stimulation (*M* = 6.31, SD = 1.95) in comparison to before stimulation (*M* = 6.61, SD = 1.77). However a closer look to the data indicates that this effect was moderated by the type of stimulation. That is, a significant interaction effect was demonstrated between the measurements and the type of stimulation (*F*(4,216) = 2.70, *p* = 0.03). A univariate analysis revealed that this interaction effect was for the tinnitus loudness (*F*(2,108) = 5.11, *p* = 0.008) as well as the tinnitus distress (*F*(2,108) = 4.18, *p* = 0.018). A simple contrast revealed that only for the tRNS condition tinnitus patients had a significant decrease on loudness (*F*(1,108) = 24.69, *p* = 0.000003) and distress (*F*(1,108) = 17.52, *p* = 0.00006) comparing post-stimulation to pre-stimulation (see Figure 1). No significant differences were obtained between the pre- and post-stimulation measurements for the tDCS condition [loudness: *F*(1,108) = 0.75, *p* = 0.39; distress: *F*(1,108) = 0.55, *p* = 0.46] and tACS condition [loudness: *F*(1,108) = 1.35, *p* = 0.25; distress: *F*(1,108) = 0.24, *p* = 0.63] on both tinnitus loudness and tinnitus distress. In addition no significant main effect was obtained for the between-subjects variable on the stimulation type (*F*(2,216 = 1.88, *p* = 0.14) for the tinnitus loudness (*F*(2,108) = 0.02, *p* = 0.98) and the tinnitus distress (*F*(2,108) = 1.49, *p* = 0.23) independent of pre- and post-stimulation. A overview can be found in Figure 1.

**Figure 2:**
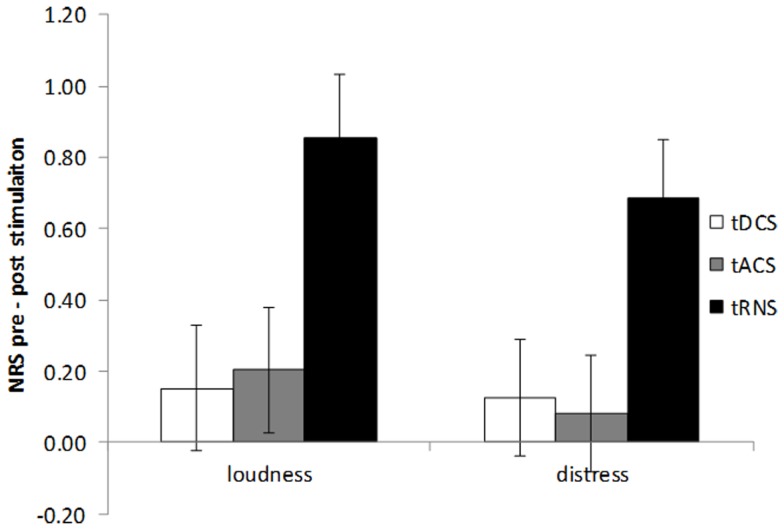
**Amount of tinnitus suppression (pre – post-stimulation) for tinnitus loudness and tinnitus distress by bilateral auditory cortex tDCS, tACS, and tRNS**. tRNS significantly improves both tinnitus loudness and tinnitus distress in comparison to tDCS and tACS.

An extra analysis on the difference scores between pre and post-stimulation for the three stimulation techniques using a multivariate ANOVA revealed a significance for the different stimulation techniques (*F*(2,216) = 2.70, *p* = 0.03). An univariate analysis revealed that this result was observed for both the loudness (*F*(2,108) = 5.11, *p* = 0.008) as well as the distress (*F*(2,108) = 4.18, *p* = 0.02) (see Figure 2). After Bonferroni correction for multiple comparisons it was revealed that there was a significant difference for tRNS in comparison to tDCS and tACS on both loudness and distress (*p* < 0.05). No significant differences were demonstrated between tDCS and tACS on both loudness and distress.

## Discussion

In the present study we tested the efficacy of different tES techniques, namely tDCS, tACS, and tRNS applied over the auditory cortex in tinnitus patients. Both loudness and distress can be modulated in tinnitus patients, but only for the tRNS condition. For tDCS and tACS no significant differences were obtained, indicating that tRNS is a more effective single session method for the transient suppression of tinnitus.

The clinical differences obtained suggest that tRNS might have a different mechanism of action in comparison to tDCS and tACS.

Previous tRNS research on healthy subjects applied to the visual areas of the brain indicated an improvement on behavioral performance in comparison to tDCS (44). These results were interpreted as a potentiation of the activity of the neural populations involved in the specific cognitive task by facilitating brain plasticity by strengthening synaptic transmission between neurons via a stochastic resonance-like phenomenon (44). Based on the idea that tRNS strengthens synaptic transmission, an increase in synchronization could be expected, which might lead to increase in the tinnitus loudness. However, the results of this study demonstrated a suppressive effect on both tinnitus loudness and distress using tRNS. One possible explanation for this seeming contradiction might be related to a brain state dependent effect, i.e., the depending on the ongoing resting state activity, analogous to what has been demonstrated for tDCS. In tDCS it has been shown that different to opposite effects can be obtained in healthy subjects in comparison to patients with a mood disorders (54, 55). That is, in healthy subjects tDCS had no effect on different mood scales, while in depressive patients it exerted an improvement (54, 55).

It is known that for healthy subjects the resting state electrical brain activity is more like a noise like signal in the auditory cortex (56–58), while for tinnitus patients it has been proposed that hyper-synchronization is present within the auditory cortex (8–11, 17, 52, 59). Hence, a possibility is that adding noise to the ongoing hyper-synchronization might disrupt this synchronization, while adding random noise to spontaneous noisy activity in healthy subjects might result in an opposite or no effect. This effect may be similar to effects of TMS and tDCS over the motor cortex for neuropathic pain; but interestingly here the effects of tRNS were larger than tDCS and tACS and in fact the only technique that induced significant effects.

Our results showed that bilateral auditory tDCS results in a small and non-significant change in tinnitus symptoms. Given that single-sided anodal stimulation of auditory cortex with cathodal stimulation of the contralateral supra-orbital area yields a significant tinnitus suppressive effect (27, 45), three potential reasons may explain the different results. First, the results of the current study may indicate that bilateral direct current stimulation of the auditory cortex may not be the optimal electrode montage for tinnitus modulation. Recent research has showed a differential effect on neural activity can be seen depending on the placement of the electrodes in tinnitus patients (60) and also on chronic pain (61). Second, the small sample size may not have yielded enough power to detect significant differences especially considering that the tDCS group was divided in half according to the hemisphere/polarity of stimulation. Third, differences in patients’ characteristics may also have resulted smaller effect sizes induced by tDCS in our study. It has been shown for chronic pain that longer and more severe diseases are associated with smaller tDCS effects (62).

Furthermore, no effect was obtained by bilateral auditory cortex tACS stimulating at the IAF. As tinnitus is associated with a decrease of alpha activity in the auditory cortex it can be expected that strengthening the IAF might reduce the tinnitus percept (16, 51). Several reasons can be proposed for the negative results obtained with tACS in this study. One possibility is that the strength of the current was too weak to induce an effect, as previous studies have used amplitudes up to 3 mA in tACS (39, 40). This can be tested by future research.

A weakness of this study is that no placebo-arm was included. However the obtained results are straightforward as the effect obtained by tRNS was clearly stronger than the effects after real tDCS and tACS even though patients do not feel a difference in sensation using the differ methods. Nevertheless, further research could benefit from using a placebo-arm as it may show that the small effects induced by tDCS and tACS may be different than placebo.

In conclusion, our findings show clear superiority effects of tRNS as compared to tACS or tDCS in suppressing tinnitus transiently when applying the electrodes over the auditory cortex bilaterally. The results of this study are important as it compares for the first time in head-to-head trial three different techniques of tES using weak currents.

## Conflict of Interest Statement

The authors declare that the research was conducted in the absence of any commercial or financial relationships that could be construed as a potential conflict of interest.

## Footnotes

^1^Congedo, M., 2002. EureKa! (Version 3.0), Knoxville, TN: NovaTech, Freeware available at http://www.NovaTechEEG.com
